# Validation study of the Italian brief version of the multidimensional jealousy scale: Psychometric properties, measurement invariance across gender, and convergent validity

**DOI:** 10.3389/fpsyg.2022.1013584

**Published:** 2022-11-22

**Authors:** Pierluigi Diotaiuti, Giuseppe Valente, Stefania Mancone, Laura Girelli, Elisa Cavicchiolo, Andrea Chirico

**Affiliations:** ^1^Department of Human Sciences, Society and Health, University of Cassino and Southern Lazio, Cassino, Italy; ^2^Department of Human, Philosophical, and Educational Sciences, University of Salerno, Fisciano, Italy; ^3^Department of Systems Medicine, Tor Vergata University of Rome, Rome, Italy; ^4^Department of Psychology of Development and Socialization Processes, Sapienza University of Rome, Rome, Italy

**Keywords:** behavioral jealousy, cognitive jealousy, emotional jealousy, gender differences, insecure attachment, multidimensional jealousy scale, Italian, romantic jealousy

## Abstract

**Introduction:**

The multidimensional jealousy scale (MJS) is among the most internationally used instruments for the assessment of jealousy in its three dimensions: cognitive, emotional, and behavioral. This study aimed to replicate the Italian validation process of the shortened MJS in order to confirm its psychometric properties and measurement invariance across gender.

**Materials and methods:**

Exploratory factor analysis (EFA) and confirmatory factor analysis (CFA) were conducted in a large sample of adults (*n* = 2,928). To reliably estimate mean differences across gender, the measurement invariance of the scale was first established by means of CFA. Convergent validity was than tested by administrating the tool to a convenient sample (*n* = 304).

**Results:**

A 15-item version of the Italian MJS was retained in its three-factor structure. The tool showed good fit with both the CFA (χ^2^ = 211.827, CFI = 0.969, TLI = 0.959, RMSEA = 0.047, RMSEA 90% CI = 0.039–0.055) and the results confirmed the strong measurement invariance of the MJS across gender. The internal consistency measures were found to be fully satisfactory. Predictive associations with constructs such as avoidance and anxiety referred to attachment in relationships (ECR-R), obsessive jealousy, depressive jealousy, jealousy associated with separation anxiety, paranoid jealousy (QUEGE), and basic self-esteem (BSE) were confirmed.

**Discussion:**

The MJS is particularly apt to collect information quickly and efficiently about jealousy in a current relationship. The multidimensional and brief structure makes it particularly suitable for preliminary screening, couple therapy assessment, and research purposes.

## Introduction

The dyadic experience (sexual or romantic) represents a significant relationship for many people and has an important and relevant effect on psychological well-being, self-esteem and emotional balance (Simpson and Overall, 2014). Jealousy can occur in all those social relationships in which the person feels a strong emotional bond with another person and this strong bond is characterized by a predominant desire for exclusivity and totality: a child who may be jealous of siblings with whom they share their parents, one’s best friend who has other friends (or sexual partner), can be examples of relational situations in which the desire for exclusivity and wholeness is very strong (Krems et al., 2021). When exclusivity and wholeness are not guaranteed, are not perceived or are in danger and threatened, jealousy is experienced, characterized by anxiety, frustration and anger.

Jealousy is a complex emotion that has the positive function of preserving a relationship and preventing others from damaging it (Chung and Harris, 2018). Like all human emotions, jealousy can be healthy or pathological, depending on the intensity with which it is manifested and the degree of control we have over feelings and related emotions and thoughts. In a recent psychological study, models seem to be moving away from dichotomizing jealousy in the sense of healthy and unhealthy in order to consider jealousy on a spectrum or continuum from normal to pathological (Lima et al., 2017; Tandler and Petersen, 2020).

Jealousy is generally defined as an aversive emotional reaction that occurs as a result of a relationship outside the partner’s dyad, which is actual imagined or believed likely to occur (Bringle, 1991; Bringle and Buunk, 1991). When the intensity is high and the emotion is out of control, it can trigger reactions of anger and aggression against the partner or loved one or even against those people whom we consider to be an obstacle or an adversary in a personal relationship that we would like to be exclusive (Petruccelli et al., 2014; Cynkier, 2018). Jealousy has both personal and social consequences, especially when the jealous person’s reaction is violent and the personal capacity for emotional regulation is diminished. Jealousy in its most violent and out of control manifestation leads to murder (Evzonas, 2018).

Most theoretical models of jealousy are multidimensional in nature (White and Mullen, 1989; Bringle, 1991) and have led to the development of a number of jealousy scales; among these, the most widely used internationally are the multidimensional jealousy scale (MJS) by Pfeiffer and Wong (1989) and the Revised Anticipated Sexual Jealousy Scale (RASJS) by Buunk (1997). Although these two instruments derive from different theoretical models and the authors also introduced different scale names for the components of jealousy to be assessed, the two inventories focus on the same three dimensions of jealousy.

According to Buunk (1997), jealousy reactions within the transactional model are determined by both endogenous variables (such as a individual’s personality, values and belief systems) and exogenous variables (such as specific situational influences).

Both the MJS (Pfeiffer and Wong, 1989) and the RASJS (Buunk, 1997) aim to assess the above subcomponents of jealousy, distinguishing normal from pathological forms of jealousy. From an evolutionary perspective, jealousy behavior is undoubtedly adaptive (Buss, 2000). As pathological, many authors consider in particular strong expressions of preventive and anxious jealousy, whereas reactive jealousy is interpreted as normal and situationally normal and adapted to the situation (Stravogiannis et al., 2018). In accordance with this, reactive jealousy occurs particularly strongly in relationships that have been characterized by a previously high partnership quality (Knobloch et al., 2001; Barelds and Barelds-Dijkstra, 2007; Diotaiuti et al., 2021a,b). Potentially pathological in nature, anxious and preemptive jealousy are particularly prevalent when they are self-generated and occur without cause (Pfeiffer and Wong, 1989; Barelds and Barelds-Dijkstra, 2007). As described in Bringle’s (1991) transactional model, anxious and preventive jealousy, in contrast to reactive jealousy, are more strongly determined by endogenous active factors such as pathological traits and extreme belief systems and values. In addition, however, there are also exogenous effect factors such as a partnership which is thought to be unsatisfactory, which provides a breeding ground for feelings of mistrust, worry, and suspicion.

Whereas Pfeiffer and Wong (1989) based their construction of the MJS on White’s (1981) jealousy model and distinguished between an emotional, cognitive, and behavioral jealousy, Buunk (1997) borrows the content as well as the naming of facets from Bringle’s (1991) transactional model. However, he divides suspicious jealousy into two separate components, so that in addition to the reactive jealousy facet, the RASJS is also intended to capture an anxious as well as a preventive jealousy factor. The two inventories correspond, given that the emotional jealousy facet of the MJS, like the reactive jealousy component within the RASJS, is intended to capture the dispositional extent of affective reactions that a person feels toward varying degrees of emotional or sexual infidelity on the part of a partner. The aim is to capture a form of jealousy that is characterized by a high degree of perceived obligation to control or prevent even innocent, superficial contact of the partner with a person of the opposite sex. In addition, this subfacet asks individuals about detective behaviors, such as how much they spy on their partner.

According to MJS, the cognitive manifestations of jealousy include suspicions, ideas, and anxieties about the partner’s possible attraction to another person, as well as catastrophic anticipation and unpleasant personal thoughts. Emotional jealousy, on the other hand, is a collection of predicted affective responses to threats, such as fear, sadness, rage, envy, and emotional dependency, which influences how people communicate and deal with jealously (Wegner et al., 2018; Diotaiuti et al., 2020, 2022). Behavioral jealousy is defined as the visible display of jealousy through behaviors that are used to validate the possibility of deception and to raise inquisitive concerns (Pfeiffer and Wong, 1989).

Over the years, several studies have analyzed the psychometric properties of the MJS in different cultural context. Elphinston et al. (2011) were the first to conduct a proper validation of the scale: results of a confirmatory factor analysis (CFA) conducted on an Australian sample of 127 participants (M age = 20.55 years, SD = 4.30, age range 17.5 – 49.83 years, 68.5% females) supported a 17-item version of the scale with three factors representing cognitive, emotional, and behavioral jealousy. The psychometric properties of the scale were also examined in a Serbian sample of 500 participants (age range 18 – 40, 79% female) and the results of the CFA corroborated the three-factor structure of the original 24-item instrument (Tošić-Radev and Hedrih, 2017). Brassard et al. (2019) examined the psychometric properties of the scale in a Canadian French sample of 381 participants (M age 34,31 years; 58,8% females) and the results of the CFA supported the three-factor structure of a 15-item version of the measure. Furthermore, the scale, along with its three dimensions, has proved to have good reliability (Cronbach’s alpha coefficient of 0.83–0.94) also in an Iranian sample (Rahimi and Sanatnama, 2021), although a CFA was not conducted in this sample.

In the Italian context, at the moment jealousy is assessed only through the use of the specific instrument of the QUEGE (Marazziti et al., 2010), mostly used in clinical settings for the assessment of pathological aspects in the management of the couple relationship. A further contribution of the Italian validation of the brief version of the MJS adapted for a measure in non-heterosexual couples as well could be very useful. So far, only one study attempted a first validation of the MJS in the Italian context (Tani and Ponti, 2016). In this study, confirmatory factor analyses supported the three-factor structure of romantic jealousy using a very small sample of university students. Therefore, the aim of the present study was to present a confirmatory model of the instrument accompanied by the measure of factorial invariance and convergent validity through an extended number of Italian participants. In Study 1 we examined the psychometric properties and measurement invariance across gender of the scale. The differences in jealousy across gender were examined by means of latent factor mean difference test.

Subsequently, in a second study (Study 2), we have tested the convergent validity of the scale by comparing it with the following conceptual frameworks: avoidance and anxiety referred to attachment in relationships (ECR-R, Fraley et al., 2000), obsessive jealousy, depressive jealousy, jealousy associated with separation anxiety, paranoid jealousy (QUEGE, Marazziti et al., 2010), basic self-esteem (BSE, Forsman and Johnson, 1996). As a result, we also proposed the following hypotheses: the greater the jealousy measured with MJS, the higher the Quege scoring; the higher the anxiety and the lower the avoidance referred to attachment in relationships, and the lower the basic self-esteem would be.

## Materials and methods

### Linguistic procedures

According to the EORTC translation rules, the MJS was translated using the original scale while translating it both forward and backward (Dewolf et al., 2009). Two Italian translators completed the forward translation independently and worked out any inconsistencies between the two versions. Two English translators separately back-translated the measure after receiving the reconciled Italian version. Any differences were discussed and resolved, and changes were made to the MJS to account for any rewording in order to improve the items’ conceptual relevance and comprehension. Since in the original scale by Pfeiffer and Wong (1989) several items used the wording “person of the opposite sex,” foreshadowing an administration of the instrument intended for individuals with heterosexual orientation, it was deemed appropriate to replace the above expression in the items with the more generic formula “another person,” in order to extend the use of the scale without limitations related to sexual orientation. Finally, a small focus group of ten people was formed and constructed to include people from three different age groups (20–30; 31–40; 41–50), males and females, and individuals with low, medium, and high educational qualifications. Following the administration of the MJS scale, a discussion of each item revealed no issues of comprehensibility or literacy disparities.

### Study 1

#### Participants and administration

The sample size for this study was determined by the ability to demonstrate a satisfactory fit of MJS, which started with a translation of the entire English survey’s three-factor model and its 24 manifest variables. Using the root-mean-square error of approximation (RMSEA) as the measure of model fit, a sample size of at least 240 participants gives 90% power to test RMSEA 0.05 when RMSEA = 0.08 and a significance level of 0.05 (MacCallum et al., 1996). Participants were recruited by sending a contact to university students in central Italy, outlining the study’s goals and purpose. Each student was asked to recruit four friends who had been in a romantic relationship for at least 2 months to take part in the study. Subjects were instructed to click on a URL provided in the same notice, fill out the form, and then telematically and digitally submit their responses. Participants were guaranteed anonymity as well as the usage of aggregate data for research purposes. A total of 4,500 emails with contact information were sent. In terms of the drop-out rate, 88 people dropped out after starting to fill it out, resulting in a total of 2,928 completed questionnaires (1,388 males and 1,470 females with an average age of 30.38 and SD = 11.92).

#### Measures used in study 1

Multidimensional jealousy scale (MJS, Pfeiffer and Wong, 1989): 24 items articulated into three factors (8 items per factor): *Cognitive*, *Emotional*, *Behavioral*. Cognitive Jealousy refers to the frequency, using a scale from 1 (always) to 7 (never), with which a person suspects and worries about a partner’s interest in a rival and the interest received from a rival. Emotional jealousy is defined as the degree of annoyance, assessed with a scale from 1 (I am very pleased) to 7 (I am very upset), that a person experiences when exposed to a situation that evokes jealousy. Behavioral jealousy refers to the frequency, using a scale from 1 (never) to 7 (always), with which a person engages in protective and investigative behaviors such as asking questions or surveying their partner. Scores across items are summed to provide assessment of three main dimensions of jealousy: cognitive, emotional, and behavioral.

#### Statistical analysis

Preliminary analyses were performed. To assess the dimensionality of the MJS, a first CFA with 24 items and three dimensions was performed. After the evaluation of this first model, the sample was randomly divided into two subsamples, each one made of 1,464 participants. An Exploratory Factor Analysis (EFA) was conducted on the first subsample with maximum likelihood estimation and promax rotation, using the software IBM SPSS Statistics version 26 Subsequently, by using the AMOS 5 program, a confirmatory factor analysis (CFA) was carried out on the second subsample with the maximum likelihood (ML) estimation. We chose to use ML estimation instead of weighted least squares means and variance adjusted (WLSMV) because previous studies have suggested to choose this estimation when the number of response category is >5 (Rigdon, 1998; Raykov, 2012; Morin et al., 2020) and in the present study the MJS is evaluated on a 7-point scale. In line with the theoretical expectations, we tested a model consisting of three correlated factors (i.e., Cognitive, Emotional, and Behavioral Jealousy) and chi-square test statistic, CFI (Comparative Fit Index), TLI (Tucker–Lewis Index), and RMSEA (Root-Mean-Square Error of Approximation) were used as relevant fit indicators to test the adequacy of the CFA model, as suggested by technical literature (Teo, 2010), with CFI and TLI > 0.95 and RMSEA < 0.06 as excellent model fit indicators (Yu, 2002). Item statistics and internal consistency were also analyzed. Then, the measurement invariance of the scale across gender was examined by means of a series of multigroup CFAs on the whole sample, imposing increasingly restrictive equality constraints on the model’s parameters (van de Schoot et al., 2012) and in each step of the analysis the fit of the nested models was compared using the change in CFI, and RMSEA (−0.01 for ΔCFI and 0.01 for ΔRMSEA, Putnick and Bornstein, 2016). Once the configural, metric and scalar invariance was assessed, the group means on the three latent factors (i.e., Cognitive, Emotional, and Behavioral Jealousy) were compared.

#### Results

##### Preliminary analyses

The procedure for standardizing the variables was used to verify the assumptions of univariate and multivariate normality, erasing outlier cases with values greater than 3, and then, following the calculation of the Mahlanobis Distance, the multivariate outlier cases with D^2^ greater than the critical value, calculated by using the chi-square reference distribution (level *p* < 0.001) with *p* degrees of liberty equal to the number of variables. The Mardia Index (average of the squares of the Malhanobis Distances) calculation yielded a coefficient (180.46) below the upper bound (195).

##### Dimensionality of the multidimensional jealousy scale

A confirming analysis (CFA) was used to evaluate the scale’s metric properties and determine whether Pfeiffer and Wong’s (1989) three-dimensional model was accurate (1989). Table 1 presents descriptive statistics and factor loadings for the 24 items of the scale.

**TABLE 1:** Descriptive statistics and factor loadings of the Italian multidimensional jealousy scale (MJS) (*N* = 2,928).

Item	M	SD	Bootstrap CI 95%	Skewness	SE	Kurtosis	SE	Factor 1	Factor 2	Factor 3
Item 1	6.18	1.24	(6.14–6.22)	–1.125	0.045	1.136	0.090	–0.641	0.138	–0.062
Item 2	5.22	1.80	(5.16–5.29)	–0.758	0.045	–0.482	0.090	0.689	0.160	–0.024
Item 3	5.45	1.64	(5.40–5.51)	–0.942	0.045	0.096	0.090	0.687	0.010	–0.033
Item 4	6.38	1.07	(6.34–6.42)	–1.312	0.045	1.127	0.090	–0.627	0.211	–0.086
Item 5	5.31	1.76	(5.25–5.38)	–0.812	0.045	–0.347	0.090	0.712	0.093	–0.115
Item 6	5.38	1.73	(5.31–5.44)	–0.916	0.045	–0.115	0.090	0.759	0.136	–0.067
Item 7	6.53	0.86	(6.49–6.56)	–1.179	0.045	1.049	0.090	–0.559	0.254	–0.126
Item 8	5.65	1.86	(5.58–5.72)	–1.154	0.045	0.404	0.090	0.493	0.028	–0.021
Item 9	4.93	1.53	(4.87–4.98)	–0.290	0.045	–0.511	0.090	0.096	0.581	0.116
Item 10	5.25	1.45	(5.20–5.30)	–0.534	0.045	–0.317	0.090	0.068	0.708	0.050
Item 11	5.26	1.46	(5.21–5.31)	–0.608	0.045	–0.169	0.090	0.033	0.714	0.057
Item 12	5.82	1.28	(5.77–5.86)	–0.873	0.045	–0.094	0.090	–0.022	0.718	–0.023
Item 13	6.34	1.04	(6.30–6.38)	–1.132	0.045	1.200	0.090	–0.052	0.678	–0.130
Item 14	5.88	1.44	(5.82–5.93)	–1.185	0.045	0.618	0.090	–0.041	0.634	–0.002
Item 15	6.06	1.30	(6.01–6.11)	–1.138	0.045	0.522	0.090	–0.057	0.628	–0.048
Item 16	4.80	1.66	(4.74–4.76)	–0.347	0.045	–0.587	0.090	–0.051	0.468	0.187
Item 17	2.31	1.86	(2.25–2.38)	1.057	0.045	0.290	0.090	–0.086	–0.080	0.788
Item 18	2.49	1.94	(2.42–2.56)	1.085	0.045	–0.123	0.090	–0.021	–0.064	0.843
Item 19	3.35	2.01	(3.28–3.42)	0.379	0.045	–1.076	0.090	0.118	0.091	0.529
Item 20	3.32	2.05	(3.24–3.39)	0.394	0.045	–1.137	0.090	0.053	0.170	0.585
Item 21	3.23	2.03	(3.15–3.30)	0.454	0.045	–1.097	0.090	–0.004	0.115	0.752
Item 22	3.46	2.01	(3.38–3.53)	0.319	0.045	–1.153	0.090	0.029	0.118	0.718
Item 23	2.91	1.94	(2.84–2.98)	0.728	0.045	–0.685	0.090	–0.046	0.006	0.712
Item 24	2.26	1.81	(2.20–2.32)	1.138	0.045	0.624	0.090	–0.025	–0.138	0.829

M, mean; SD, standard deviation; CI, confidence interval; SE, standard error; Factor 1 = Cognitive Jealousy; Factor 2 = Emotional Jealousy; Factor 3 = Behavioral Jealousy. Extraction method: Maximum Likelihood; Rotation method: Promax with Kaiser normalization; Rotation converged in 5 interations.

Three factors and 24 items were taken into account in the results, but they did not adequately fit the data. Therefore, the original sample was randomly divided into two subsamples, each one made of 1,464 participants. Following Tables 2, 3 report descriptive statistics for the two subsamples.

**TABLE 2:** Descriptive statistics of the first subsample (*n* = 1,464).

Item	M	SD	Bootstrap CI 95%	Skewness	SE	Kurtosis	SE
Item 1	6.17	1.25	(6.10–6.23)	–1.485	0.064	1.282	0.128
Item 2	2.78	1.78	(2.69–2.87)	0.728	0.064	–0.476	0.128
Item 3	2.55	1.61	(2.46–2.63)	0.914	0.064	0.108	0.128
Item 4	6.39	1.07	(6.34–6.45)	–1.763	0.064	1.085	0.128
Item 5	2.61	1.69	(2.52–2.69)	0.867	0.064	–0.137	0.128
Item 6	2.55	1.67	(2.47–2.64)	0.949	0.064	–0.005	0.128
Item 7	6.54	0.85	(6.50–6.59)	–1.832	0.064	1.262	0.128
Item 8	2.30	1.87	(2.20–2.40)	1.333	0.064	0.580	0.128
Item 9	4.89	1.50	(4.81–4.97)	–0.258	0.064	–0.482	0.128
Item 10	5.23	1.44	(5.16–5.30)	–0.522	0.064	–0.271	0.128
Item 11	5.20	1.53	(5.12–5.28)	–0.629	0.064	–0.146	0.128
Item 12	5.83	1.27	(5.76–5.90)	–0.836	0.064	–0.261	0.128
Item 13	6.32	1.07	(6.27–6.38)	–1.477	0.064	1.128	0.128
Item 14	5.83	1.46	(5.75–5.90)	–1.159	0.064	0.669	0.128
Item 15	6.02	1.32	(5.95–6.09)	–1.161	0.064	0.342	0.128
Item 16	4.75	1.63	(4.66–4.83)	–0.319	0.064	–0.573	0.128
Item 17	2.15	1.71	(2.06–2.24)	1.413	0.064	0.867	0.128
Item 18	2.29	1.77	(2.21–2.38)	1.253	0.064	0.446	0.128
Item 19	3.33	1.93	(3.23–3.43)	0.374	0.064	–0.996	0.128
Item 20	3.26	2.01	(3.15–3.35)	0.423	0.064	–1.071	0.128
Item 21	3.03	1.91	(2.93–3.13)	0.550	0.064	–0.897	0.128
Item 22	3.31	1.92	(3.21–3.41)	0.400	0.064	–1.012	0.128
Item 23	2.79	1.84	(2.70–2.89)	0.792	0.045	–0.465	0.128
Item 24	2.11	1.64	(2.02–2.19)	1.469	0.045	1.213	0.128

M, mean; SD, standard deviation; CI, confidence interval; SE, standard error.

**TABLE 3:** Descriptive statistics of the second subsample (*n* = 1,464).

Item	M	SD	Bootstrap CI 95%	Skewness	SE	Kurtosis	SE
Item 1	6.18	1.23	(6.08–6.27)	–1.478	0.090	1.204	0.180
Item 2	2.71	1.83	(2.58–2.85)	0.848	0.090	–0.376	0.180
Item 3	2.56	1.67	(2.44–5.68)	0.894	0.090	–0.085	0.180
Item 4	6.33	1.09	(6.25–6.41)	–1.586	0.090	1.429	0.180
Item 5	2.71	1.80	(2.58–2.85)	0.736	0.090	–0.582	0.180
Item 6	2.61	1.78	(2.48–2.74)	0.867	0.090	–0.295	0.180
Item 7	6.50	0.89	(6.43–6.56)	–1.711	0.090	1.773	0.180
Item 8	2.36	1.85	(2.24–2.50)	1.224	0.090	0.377	0.180
Item 9	4.85	1.56	(4.74–4.96)	–0.313	0.090	–0.387	0.180
Item 10	5.22	1.46	(5.12–5.33)	–0.531	0.090	–0.264	0.180
Item 11	5.26	1.38	(5.15–5.36)	–0.518	0.090	–0.209	0.180
Item 12	5.74	1.32	(5.64–5.84)	–0.912	0.090	0.070	0.180
Item 13	6.31	1.00	(6.23–6.38)	–1.372	0.090	0.943	0.180
Item 14	5.95	1.38	(5.84–6.04)	–1.162	0.090	0.402	0.180
Item 15	6.04	1.31	(5.95–6.13)	–1.171	0.090	0.270	0.180
Item 16	4.88	1.63	(4.76–5.00)	–0.402	0.090	–0.436	0.180
Item 17	2.34	1.91	(2.20–2.48)	1.284	0.090	0.365	0.180
Item 18	2.49	1.93	(2.36–2.62)	1.099	0.090	–0.074	0.180
Item 19	3.22	2.11	(3.07–3.37)	0.476	0.090	–1.094	0.180
Item 20	3.19	2.05	(3.03–3.34)	0.485	0.090	–1.037	0.180
Item 21	3.17	2.05	(3.02–3.31)	0.490	0.090	–1.069	0.180
Item 22	3.41	2.07	(3.25–3.56)	0.327	0.090	–1.188	0.180
Item 23	2.89	2.01	(2.74–3.03)	0.739	0.090	–0.749	0.180
Item 24	2.31	1.91	(2.17–2.44)	1.277	0.090	0.295	0.180

M, mean; SD, standard deviation; CI, confidence interval; SE, standard error.

The occurrence of fewer items was verified by performing an EFA with ML and items 1, 4, 7, 10, 13, 14, 18, 22, and 24 were removed because they were found to damage the fit between the model and the covariance structure. The following fit values were attained by leaving out these nine items: Kaiser-Meyer-Olkin (KMO) index score was 0.849, Chi-squared Test < 0.001; RMSEA = 0.048; RMSEA 90% 0.44–0.052; TLI = 0.950. The model matrix for the three identified factors is shown in Table 4 with saturations, and the factorial interrelationships are reported in Table 5.

**TABLE 4:** EFA pattern matrix of the MJS (15 items).

	Cognitive jealousy	Behavioral jealousy	Emotional jealousy
Item 6	**0.850**	0.027	–0.020
Item 5	**0.800**	0.066	0.031
Item 2	**0.726**	–0.036	–0.043
Item 3	**0.573**	–0.043	0.051
Item 8	**0.433**	–0.029	0.031
Item 21	–0.006	**0.762**	0.044
Item 23	0.040	**0.724**	–0.035
Item 17	0.084	**0.711**	–0.060
Item 20	–0.043	**0.683**	0.063
Item 19	–0.138	**0.600**	–0.013
Item 11	–0.007	–0.033	**0.802**
Item 12	0.017	–0.058	**0.708**
Item 9	–0.076	0.074	**0.594**
Item 15	0.036	–0.065	**0.565**
Item 16	0.082	0.115	**0.532**

Extraction Method: Maximum Likelihood. Rotation Method: Promax with Kaiser Normalization. Rotation converged in five iterations. Item number relates to Pfeiffer and Wong (1989). The bold values represent the factor where each item saturates.

**TABLE 5:** Factor inter-correlations.

	Cognitive	Behavioral	Emotional
Cognitive jealousy	1		
Behavioral jealousy	−0.265**	1	
Emotional jealousy	−0.097**	0.355**	1

**Correlation is significant at the 0.01 level (2-tailed).

**FIGURE 1:**
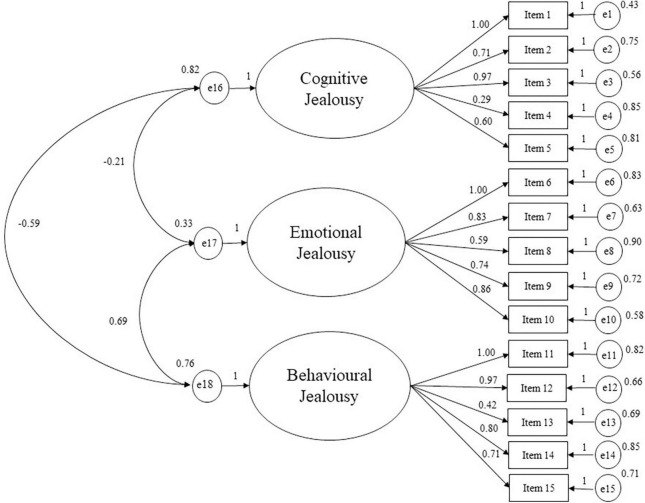
Path diagram of the confirmatory analysis concerning multidimensional jealousy scale (MJS) (15 items). X^2^/df = 2.61; RMSEA = 0.047; RMSEA 90% CI = 0.039–0.055; TLI = 0.959; CFI = 0.969.

Then, the CFA conducted on the second subsample showed that the model with three related factors and 15 items indicated acceptable fit with the empirical data (see Figure 1): Chi-square = 211.827; chi^2^/df = 2.61; CFI = 0.969; TLI = 0.959; RMSEA = 0.047 and RMSEA 90% CI [0.039–0.055]. The first factor measures *Cognitive Jealousy* (5 items); the second factor measures *Emotional Jealousy* (5 items); the third factor measures *Behavioral Jealousy* (5 items).

##### Item statistics and internal consistency

The following Table 6 shows item statistics and internal consistency of the 15-item version of the scale. All of the items showed some ceiling effects, and these ranged from 21.1% (item 10) to 56.6% (item 9). Item 9, which is the one that aroused the most jealousy, had a mean score of 6.06, whereas item 11, which is the one that aroused the least jealousy, had a mean score of 2.31. The overall mean score was 54.94 (15–105) with a SD of 25.88. MJS showed acceptable internal consistency: for Cognitive Jealousy α was 0.80 and ω = 0.81; for Emotional Jealousy α was 0.77 and ω = 0.78 and finally for Behavioral Jealousy α was 0.83 and ω = 0.83.

**TABLE 6:** The item statistics and reliability of MJS.

	Mean (SD)	Skewness	Kurtosis	Item-total correlation	Cronbach’s alpha if item drops	Response							Cronbach’s alpha [95% IC]	McDonald’s omega [95% IC]	Gutmann’s lamda [95% IC]	Average inter-item correlation [95% IC]

						Never (ceiling effect)	2	3	4	5	6	All the time (floor effect)				
Item 1	2.78 (1.80)	0.76	–0.48	0.64	0.75	35.1	18.0	14.0	14.3	8.6	5.3	4.7	0.80 [0.793/0.815]	0.81 [0.801/0.822]	0.79 [0.774/0.799]	0.454 [0.435/0.472]
Item 2	2.54 (1.63)	0.93	0.06	0.54	0.78	36.9	20.9	15.1	14.0	6.7	3.3	3.1				
Item 3	2.69 (1.75)	0.80	–0.037	0.66	0.74	36.5	18.6	13.5	14.4	8.3	4.7	4.0				
Item 4	2.61 (1.72)	0.90	–0.16	0.71	0.73	37.0	20.6	14.0	12.4	7.9	4.4	3.8				
Item 5	2.35 (1.86)	1.25	0.40	0.41	0.80	53.8	12.4	9.0	10.6	4.5	3.1	6.5				

						**Very pleased (floor effect)**	**2**	**3**	**4**	**5**	**6**	**Very upset** **(ceiling effect)**				

Item 6	4.93 (1.53)	–0.29	–0.51	0.54	0.73	2.2	3.9	8.9	27.6	20.6	15.4	21.5	0.77 [0.760/0.785]	0.78 [0.764/0.789]	0.75 [0.731/0.760]	0.410 [0.391/0.428]
Item 7	5.26 (1.46)	–0.61	–0.17	0.65	0.69	1.6	2.7	6.6	19.7	21.5	22.3	25.6				
Item 8	5.82 (1.28)	–0.87	–0.09	0.57	0.73	0.0	1.4	3.8	12.6	17.2	23.7	41.2				
Item 9	6.06 (1.30)	–1.24	0.52	0.48	0.75	0.0	1.6	3.6	10.8	11.7	15.7	56.6				
Item 10	4.80 (1.66)	–0.35	–0.58	0.51	0.75	3.9	5.9	9.1	26.1	18.7	15.1	21.1				

						**Never** **(ceiling effect)**	**2**	**3**	**4**	**5**	**6**	**All the time** **(floor effect)**				

Item 11	2.31 (1.86)	1.26	0.29	0.58	0.80	55.6	12.9	7.7	7.5	6.4	4.7	5.1	0.83 [0.816/0.836]	0.83 [0.819/0.839]	0.80 [0.784/0.810]	0.487 [0.466/0.507]
Item 12	3.35 (2.01)	0.38	–1.07	0.57	0.81	26.6	14.2	14.3	15.4	11.3	8.3	9.9				
Item 13	3.31 (2.05)	0.39	–1.13	0.69	0.77	29.3	14.2	11.3	15.2	11.6	8.1	10.4				
Item 14	3.22 (2.03)	0.45	–1.09	0.65	0.78	30.1	15.7	11.6	13.4	11.5	8.6	9.1				
Item 15	2.91 (1.94)	0.73	–0.68	0.62	0.79	34.4	18.1	13.0	12.1	8.5	6.6	7.3				
Overall	54.94 (25.88)	0.29	–0.30										0.81 [0.805/0.824]	0.82 [0.806/0.826]	0.85 [0.84/0.861]	0.223 [0.211/0.52]

The score of items 1, 2, 3, 4, 5 have been reversed. The stem for items 1, 2, 3, 4, 5 (cognitive jealousy) is: “How often do you have the following thoughts about X?”; The stem for items 6, 7, 8, 9, 10 (emotional jealousy) is: “How do you react emotionally in the following situations?”; The stem for items 11, 12, 13, 14, 15 (behavioral jealousy) is: “How often do you perform the following behaviors?”

##### Measurement invariance of the multidimensional jealousy scale across gender

Furthermore, the measurement invariance of the MJS across gender was established in four different steps (configural, metric, scalar and strict invariance) by imposing increasingly restrictive model constraints. The results of the multigroup CFAs across gender are presented in Table 7. They showed that the MJS had strong measurement invariance across gender and that the fit of the one-dimensional model for male and female was excellent.

**TABLE 7:** Goodness-of-fit indices for invariance of the MJS across gender.

	χ^2^	*df*	Δχ^2^	Δ*df*	CFI	TLI	RMSEA	ΔCFI	ΔTLI	ΔRMSEA
Configural	341.340*	158	–	–	0.957	0.943	0.056	–	–	–
Metric	354.194*	170	12.854	12	0.957	0.947	0.054	0.000	0.004	–0.002
Scalar	395.435*	182	41.241	12	0.950	0.943	0.056	–0.007	–0.004	0.002
Strict	534.784*	205	139.349	23	0.941	0.935	0.064	–0.009	–0.008	0.008

*df* = degrees of freedom; χ^2^ = Chi square; Δχ^2^ = difference in Chi square; Δdf = difference in degrees of freedom; CFI = comparative fit index; TLI = Tucker–Lewis index; RMSEA = root mean square error of approximation; ΔCFI = difference in comparative fit index; ΔTLI = difference in Tucker–Lewis index; ΔRMSEA = difference in root mean square error of approximation.

*p < 0.001.

##### Latent mean differences across gender

These findings suggest that it is possible to compare the latent means across gender. The latent mean values were fixed to zero for females and, as can be seen in the following Table 8, males showed higher latent mean values of Jealousy in this study.

**TABLE 8:** Results of the latent factor mean differences tests.

Variable	Factor	Mean	SE	CR	*P*
Gender (male)*	Cognitive jealousy	0.97	0.10	9.32	<0.001
	Emotional jealousy	0.96	0.11	8.33	<0.001
	Behavioral jealousy	1.16	0.12	9.53	<0.001

SE, standard error; CR, critical ratio; *Reference variable is female.

##### The final Italian version of the multidimensional jealousy scale

Table 9 shows the English and Italian versions of the MJS, as well as the item groupings based on respective factors.

**TABLE 9:** Multidimensional jealousy scale (MJS).

English version	Italian version
	
Please think of a person with whom you are having or have had a strong romantic/love relationship. The person is referred to as X in this questionnaire. Please rate your response to the following questions	Per favore, pensa ad una persona con cui hai o hai avuto una intensa relazione d’amore. Di seguito questa persona viene indicate con una X. Per favore indica la Tua risposta alle seguenti domande
***How often do you have the following thoughts about X?***	***Quanto spesso hai i seguenti pensieri riguardo a X?***
1. I am worried that someone may be chasing after X (CJ).	1. Sono preoccupato/a che qualcuno vada dietro a X.
2. I suspect that X may be attracted to someone else (CJ).	2. Sospetto che X possa essere attratto da qualcun altro/a.
3. I think that someone else might be romantically interested in X (CJ).	3. Penso che un’altra persona possa essere innamorata di X.
4. I am worried that someone else is trying to seduce X (CJ).	4. Sono preoccupato/a che un’altra persona stia cercando di sedurre X.
5. I suspect that X is crazy about someone else (CJ).	5. Sospetto che X vada pazzo/a per qualcun altro/a.
***How would you emotionally react to the following situations?***	***Come reagisci emotivamente nelle seguenti situazioni?***
6. X comments to you on how great looking a particular person is (EJ).	6. X commenta con te quanto sia attraente una particolare persona.
7. X smiles in a very friendly manner at someone else (EJ).	7. X sorride in un modo molto confidenziale a un’altra persona.
8. Someone else is trying to get close to X all the time (EJ).	8. Qualcun altro/a cerca ogni scusa per avvicinarsi a X.
9. X hugs and kisses someone else (EJ).	9. X abbraccia e bacia un’altra persona.
10. X works very closely with another person (in school or office) (EJ).	10. X lavora molto vicino (o studia insieme) a un’altra persona.
***How often do you engage in the following behaviors?***	***Quanto spesso metti in atto i seguenti comportamenti?***
11. I look through X’s drawers, handbag, or pockets (BJ).	11. Rovisto nei cassetti, borse o tasche di X.
12. I question X about previous or present romantic relationships (BJ).	12. Faccio domande a X su sue presenti o precedenti relazioni sentimentali.
13. I say something nasty about someone else if X shows an interest in that person (BJ).	13. Dico qualcosa di scortese riguardo qualcun altro/a se X mostra un interesse per lui/lei.
14. I question X about his or her telephone calls (BJ).	14. Faccio domande a X riguardo alle sue telefonate.
15. I join in whenever I see X talking closely to someone else (BJ).	15. Mi unisco alla conversazione ogni volta che vedo X parlare a stretto contatto con qualcun altro/a.

CJ, cognitive jealousy; EJ, emotional jealousy; BJ, behavioral jealousy.

As far as the scoring of the instrument is concerned, the 15 items in total are distributed over three factors that comprise five items each. Scores for each item range from 1 to 7. The scoring calculation generates distinct measurements for each factor by adding the scores of the component items. The factors’ combined scores can range from 5 to 35, while the total score ranges from 15 to 105. In calculating the total score, the Cognitive Jealousy subscale should be reversed.

### Study 2

#### Participants and administration

The convergent validity was examined using an additional convenient sample of 304 people, 128 males (42%), M_age_ 24.74, and SD = 7.31, all of whom were recruited online. In this instance, the prerequisite for inclusion was not participating in the prior administration. The recruitment process was conducted in the months of January and February 2022.

#### Measures used in study 2

(a) The MJS as resulted in Study 1 (three-factors, 5 items each) was used in this additional sample of people. Scores across items are summed to provide three dimensional assessments (cognitive, emotional, behavioral) and a total score of Jealousy. Reliability measures for this study were, respectively for Cognitive Jealousy: α = 0.76 [CIs 95%0.691;0.809]; ω = 0.78; [CIs 95%0.724;0.837], for Emotional Jealousy: α = 0.82 [CIs 95%0.758;0.861]; ω = 0.82; [CIs 95%0.764;0.858], and for Behavioral Jealousy: α = 0.70 [CIs 95%0.673;0.768]; ω = 0.72; [CIs 95%0.651;0.788]. Total score of MJS showed the following: α = 0.79 [CIs 95%0.733;0.842]; ω = 0.78; [CIs 95%0.724;0.816].

(b) *Experiences in Close Relationships - Revised* (ECR-R, Fraley et al., 2000; Busonera et al., 2014): the ECR-R is a revised measure of the Experiences in Close Relationships questionnaire (ECR; Brennan et al., 1998) designed to assess attachment-related anxiety and attachment-related avoidance toward romantic partners (Fraley et al., 2000). It consists of two subscales of 18 items each, assessing Intimacy Avoidance (or discomfort with closeness), and Rejection or Abandonment Anxiety (associated with jealousy and concern about attachment in relationships with romantic partners), respectively. Participants rate how much they agree or disagree with each of the 18 statements on a 7-point Likert scale ranging from 1 (strongly disagree) to 7 (strongly agree). Scores across items are summed to provide two assessments: attachment-related anxiety and attachment-related avoidance toward partners. Reliability measures for this study were, respectively for attachment-related anxiety: α = 0.74 [CIs 95%0.671;0.802]; ω = 0.75; [CIs 95%0.685;0.811], and for attachment-related avoidance: α = 0.70 [CIs 95%0.631;0.763]; ω = 0.72; [CIs 95%0.659;0.785].

(c) The *Jealousy Questionnaire* (QUEGE, Marazziti et al., 2010): a self-report instrument that consisted of 30 items that explore the presence, frequency, and duration of jealousy-related feelings and behaviors. Items are scored from one to four where 1 denotes the absence of jealousy-related behaviors/feelings and 4 denotes the highest frequency (or duration) of behaviors/feelings. The instrument measures four subtypes of jealousy: obsessive jealousy, which is characterized by involuntary and constant feelings of jealousy, of whose excessiveness the individual is aware, but that he is not able to contain; depressive jealousy, characterized by a sense of inadequacy toward the partner and the inability to trust the partner, considering the betrayal with other rivals inevitable; jealousy associated with separation anxiety, characterized by the inability to accept the prospect of a loss, with the perception of unsustainability. Consequently, the relationship becomes an addiction in which the person constantly requires the closeness of the partner and the possibility of losing him/her is repeatedly and dramatically analyzed. Paranoid jealousy, which is characterized by extreme distrust and suspicion with controlling and guessing behaviors in relation to the partner and any potential rival. Scores across items are summed to provide four assessments and a total score. Reliability for this study, respectively: obsessive jealousy α = 0.86 [CIs 95%0.825;0.893]; ω = 0.87; [CIs 95%0.834;0.899], depressive jealousy α = 0.83 [CIs 95%0.791;0.869]; ω = 0.84; [CIs 95%0.802;0.878], paranoid jealousy α = 0.75 [CIs 95%0.681;0.807]; ω = 0.76; [CIs 95%0.695;0.818], separation anxiety α = 0.78 [CIs 95%0.716;0.831]; ω = 0.78; [CIs 95%0.727;0.839], QUEGE total score α = 0.75 [CIs 95%0.681;0.807]; ω = 0.76; [CIs 95%0.695;0.818].

(d) *Basic Self-Esteem Scale* (BSE, Forsman and Johnson, 1996; Italian validation, Forsman et al., 2003): a unidimensional self-report instrument composed of 22 items with a Likert scale from 1 (totally disagree) to 5 (totally agree), for measuring basic self-esteem, i.e., the type of self-esteem that develops during childhood, through the child’s relationships with significant figures, and that constitutes in adults a rather stable personality characteristic, independent of skills and achievements, or the approval of others. Scoring is carried out by summing the scores of the component items. Reliability for this study: α = 0.90 [CIs 95%0.873;0.918]; ω = 0.90; [CIs 95%0.881;0.925].

#### Statistical analysis

A further confirmatory factor analysis (CFA) was carried out on this additional sample with the maximum likelihood (ML) estimation. The Pearson’s correlations were computed between the MJS and several other scales (ECR-R, QUEGE, BSE) to determine convergent validity. Associations among variables were evaluated also in terms of effect size, following Cohen’s (1988) guidelines, whereby values of 0.1, 0.3, and 0.5 reflect a small, medium, and large effect size, respectively.

#### Results

The CFA showed that the model with three related factors and 15 items indicated good fit with the empirical data: Chi-square = 107.601; chi^2^/df = 1.31; CFI = 0.960; TLI = 0.950; RMSEA = 0.045 and RMSEA 90% CI [0.015–0.067]. The internal consistency of the two samples is compared in Table 10 along with their respective confidence intervals. For these convergent administrations, McDonald’s and Alpha coefficients ranged from 0.78 to 0.88 (Cognitive), from 0.80 to 0.83 (Emotional), and from 70 to 0.72 (Behavioral), respectively.

**TABLE 10:** Internal reliabilities of the two samples.

		Sample 1 (*n* = 1,464)				Sample 2 (*n* = 304)		
				
	α	C.I.	ω	C.I.	α	C.I.	ω	C.I.
Cognitive jealousy	0.80	[0.79, 0.81]	0.81	[0.80, 0.82]	0.76	[0.69, 0.81]	0.78	[0.72, 0.83]
Emotional jealousy	0.77	[0.76, 0.78]	0.78	[0.76, 0.79]	0.82	[0.76, 0.86]	0.82	[0.76, 0.86]
Behavioral jealousy	0.83	[0.82, 0.84]	0.83	[0.82, 0.84]	0.70	[0.62, 0.77]	0.72	[0.65, 0.79]
Total *MJS*	0.81	[0.81, 0.82]	0.82	[0.81, 0.83]	0.79	[0.73, 0.84]	0.76	[0.72, 0.81]

α = Cronbach’s alpha; ω = McDonald’s omega; C.I. = 95% Confidence interval.

Regarding the hypotheses stated about these associations, as shown in Table 11, the obtained results have substantially confirmed the hypothesized directions. In terms of effect size and taking Cohen’s (1988) indications into account, the relationship between the total measure of jealousy carried out with the MDJ and that using the Quege showed a large effect size (>0.5), fully confirming the convergence between the two instruments. As hypothesized, a positive relationship also emerged with abandonment anxiety (effect size medium: 0.39) and an inverse relationship (effect size small: −0.21) with respect to Intimacy Avoidance. Finally, the inverse relationship with the self-esteem measure (BSE) was confirmed reporting a medium effect size (−0.32). Correlations between MJS and ECR, BSE, QUEGE are shown in Table 11.

**TABLE 11:** Bivariate correlations between MJS and ECR-R, BSE, SFCS, QUEGE.

	COG-J	EMO-J	BEH-J	TOT-J	AA	IA	BSE	OBS	PAR	SEP	DEP	TOT-Q
Cognitive jealousy (MJS)	1											
Emotional jealousy (MJS)	0.164	1					*					
Behavioral jealousy (MJS)	0.298	0.282	1									
Total jealousy (MJS)	0.712	0.701	0.701	1								
Abandonment Anxiety (ECR)	0.436	0.175	0.219	0.399	1							
Intimacy avoidance (ECR)	–0.307	0.048	–0.192	–0.210	–0.303	1						
Basic self-esteem (BSE)	–0.230	–0.031	–0.222	–0.223	–0.611	0.391	1					
Obsessive jealousy (QUEGE)	0.408	0.245	0.275	0.443	0.710	–0.152	–0.477	1				
Paranoid jealousy (QUEGE)	0.397	0.108	0.383	0.412	0.423	–0.403	–0.375	0.482	1			
Separation jealousy (QUEGE)	0.477	0.231	0.504	0.564	0.548	–0.290	–0.352	0.565	0.554	1		
Depressive jealousy (QUEGE)	0.312	0.141	0.350	0.372	0.600	–0.212	–0.616	0.561	0.516	0.538	1	
Total jealousy (QUEGE)	0.489	0.230	0.462	0.553	0.720	–0.310	–0.574	0.824	0.749	0.822	0.828	1

COG-J, Cognitive Jealousy; EMO-J, Emotional Jealousy; BEH-J, Behavioural Jealousy; AA, Abandonment Anxiety; IA, Intimacy Avoidance; BSE, Basic Self-Esteem; OBS, Obsessive Jealousy; PAR, Paranoid Jealousy; SEP, Separation Jealousy; DEP, Depressive Jealousy; TOT-Q, Total Jealousy. Any correlation above *r* = 0.16 was significant at *p* < 0.05 (two-tailed).

## Discussion

This study aimed to develop and present a validation study for the Italian version of the *Multidimensional Jealousy Scale* (MJS). The analyses conducted resulted in the formulation of a 15-item scale that independently converges on three factors. Therefore, the original three-factor model by Pfeiffer and Wong (1989) was confirmed, but adequate fit of the model required the exclusion of three items from each subscale.

The first factor measures the person’s frequency of suspicions and worries regarding their partner’s interest in a rival, and the interest received from a rival. The convergent validity analysis indicated the significant positive association with Separation Anxiety, Obsessive Jealousy, and the negative association with Avoidance of Intimacy and Basic Self-Esteem. Correlations indicate the strong need to maintain physical contact and closeness accompanied by the obsessive presence of feelings and thoughts associated with fear of abandonment. The study by Kılıç and Altınok (2021) illustrates how the obsessive tendency increases brooding activity which in turn reinforces feelings of jealousy, resulting in a deterioration of satisfaction in the intimate relationship. Several studies have confirmed that a person’s low self-esteem or a perceived threat to their self-esteem may be predictive components of the pervasiveness of thoughts oriented toward continued distrust and suspicion of the partner (DiBello et al., 2015; Verrastro et al., 2016; Chin et al., 2017; Martínez-León et al., 2017; Răşcanu, 2017; Tortoriello et al., 2017; Arcinas et al., 2021). The relationship between obsessive jealousy and pathological affective dependence has also been highlighted by previous research (Petruccelli et al., 2014; Costa et al., 2015; Stravogiannis et al., 2018; Buunk and Dijkstra, 2021).

The second factor gauges how upset a person becomes when confronted with jealousy-inspiring circumstances. The convergence associations that emerged concern separation anxiety and the obsessive component of thoughts and feelings, which the person is aware of but cannot control. The work of Dominguez-Pereira et al. (2019) provides a useful transactional interpretation of the difference between the cognitive component of romantic jealousy (referred to as suspicious jealousy by the authors) and that characterized by personal reactivity to jealousy-evoking situations. Consequently, the transactional model of jealousy conceives romantic jealousy as a social construct that is embedded within a social context. Romantic jealousy is regarded as resulting from a transaction between the person and the social environment in which both the stimulus and the person contribute to the experience of jealousy. Suspicious jealousy involves primarily thoughts, behaviors, and feelings that are usually experienced in the absence of any major jealousy-evoking event. Reactive jealousy occurs when concrete transgressions violate critical aspects of the relationship, such as the expectation of sexual exclusivity. Thus, reactive jealousy is expected to be a direct response to the identification of concrete events that might threaten the relationship. According to Rydell and Bringle (2007), reactive jealousy has the purpose of protecting the relationship from threats caused by rivals, whereas suspicious jealousy seems to be a more complex phenomenon with different and more complex underlying mechanisms.

The experience and responses are determined by individual attachment styles (Guerrero, 1998; Dandurand and Lafontaine, 2014; Martínez-León et al., 2017). People with insecure attachment styles may be more prone to interpret situations as threatening to their relationships and experience more jealousy than people with secure attachment styles (Guerrero, 1998). As reported in Dominguez-Pereira et al. (2019), the empirical evidence suggests a positive association between anxious attachment and reactive jealousy (Knobloch et al., 2001) and the same positive correlation between avoidant attachment and suspicious jealousy (Güclü et al., 2017).

When considering Pfeiffer and Wong’s (1989) tripartition into cognitive, emotional, and behavioral components, in studies that have previously explored the relationship with attachment styles, the strength of associations varied, with the association between attachment anxiety and cognitive and behavioral jealousy being about twice as strong as the association between attachment anxiety and emotional jealousy (Rodriguez et al., 2015; Bevan, 2017). The correlation between emotional jealousy and attachment anxiety was also less strong in our study than that manifested by the cognitive and behavioral component.

As indicated by Attridge (2013), emotional jealousy (reactive component) can also be associated with positive aspects of relationship survival, such as increased relational intimacy and loving involvement. In response to a jealous partner, one may avoid forming other relationships or no longer take his or her current partner for granted. Using the MJS, Rydell and Bringle (2007) discovered that higher levels of emotional/reactive jealousy were associated with higher levels of relationship dependence and partner trust. In contrast, the most dysfunctional aspects to the relationship are associated with high levels of cognitive and behavioral jealousy. Additionally, Rydell and Bringle (2007) have found that higher levels of suspicious jealousy (as determined by a combined index of the cognitive and behavioral subscales of the MJS) were linked to higher levels of relationship insecurity, lower levels of partner trust, and a number of individual difference measures that were negatively valued (i.e., anxious romantic attachment style and lower self-esteem).

The third factor of the scale measures the frequency with which a person engages in detective and protective behaviors, such as questioning and surveillance of their partner. People who are anxiously attached often worry that their loved ones can’t be trusted and have a severe, ongoing fear of rejection (Mikulincer and Shaver, 2003). They actively watch their romantic partner’s behavior for cues of availability (or unavailability) and they frequently interpret ambiguous behavior as a threat to the relationship. When a person perceives (real or imagined) relationship rivals as a threat, behavioral jealousy results in actions such as searching through a partner’s belongings or reading through their texts or emails.

With regard also to the behavioral component of jealousy, the correlations that emerged in our study are consistent with what was reported in the Australian validation of the scale (Elphinston et al., 2011) where there is a strong significant correlation with anxious attachment. Behavioral jealousy appeared to be the most problematic, according to Rodriguez et al. (2015), because it entails actions that are frequently regarded as abnormal or unacceptable. Negative partner-directed behaviors, such as jealously mediated increased monitoring of a partner’s Facebook activities, were linked to anxious attachment. A lack of trust in the partner, coupled with anxious attachment, may result in self-fulfilling prophecies that reinforce maladaptive beliefs and expectations regarding the partner’s level of trustworthiness. Their findings also demonstrated a correlation between increased psychological abuse and lower trust and higher attachment anxiety. In relation to responsiveness to jealousy solicitation scenarios, it must be acknowledged that the digital world has naturally amplified jealousy because a great number of activities on social networks are in the public domain and one can easily have access to a partner’s cell phone and computer. In these cases, social networking and technology play an active role in fueling the form of addiction that jealousy can create (Hira and Bhogal, 2020; Sullivan, 2021). Facebook, for example, exposes individuals to vague and ambiguous information about their partner that only increases worry and negative thoughts, turning social networks into a medium used to gather more and more information about the other (Imperato et al., 2021; Tandon et al., 2021). Social networks can have a profound influence on relationships in that they increase the amount of information individuals receive from their partner, which reveals much about their daily activities (e.g., seeing if their partner is active on social networks, seeing messages left on their own wall or that of friends, etc.); secondly, they offer a socially accepted way of checking on one’s partner, especially for those who exhibit jealousy. In this way, many people tend to check on their partner’s activities, checking their profile secretly while avoiding questioning their trust through an observable act; finally, social networks make the “health status” of one’s romantic relationship public (Bevan, 2017; Demirtaş-Madran, 2018; Spottswood and Carpenter, 2020). In our study, where the measurement invariance across gender of the MJS was tested, the comparison of the latent means of the scale showed that males tended to report higher values of jealousy than females. This, of course, is consistent with what has been pointed out by many works, which interpret the meanings of the experiences of jealousy on the one hand through evolutionary models, for which the physiological measures of pain and discomfort have shown that for men more intense jealousy is experienced in the case of fear of sexual betrayal by his partner, while for women the level of stress caused by a hypothetical sentimental betrayal is higher, the fact that their partner may be in love with another person (Buss, 2000; Edlund and Sagarin, 2017; Valentova, 2019). On the other hand, more recent studies are oriented according to a cultural perspective, whereby these differences between men and women can be explained in terms of gender norms and roles according to the ideological context in which the subjects find themselves. Culture would determine the conditions that generate jealousy and the responses expected in such situations. Jealousy should not be thought of as a static, unitary emotion, but as a multifaceted emotion in which culture plays a very important role (Pines and Friedman, 1998; Ward and Voracek, 2004; Canto Ortiz et al., 2009; Buunk et al., 2011; Zandbergen and Brown, 2015; Balzarini et al., 2021).

The instrument in this study has shown good reliability of measurement in relation to gender and the excellent fit of the three-dimensional model for male and female through the involvement of a large number of participants. The scale is particularly relevant in order to quickly and efficiently gather information about jealousy in a current relationship. The multi-dimensional and short structure makes it particularly suitable for the preliminary screening that, as pointed out by Elphinston et al. (2011), in case of particularly high scores on the cognitive scale, would lead to further investigation with a subsequent clinical assessment. Compared to previous international validations, the Italian version presents an adaptation in the formulation of items, which makes it possible to administer to non-heterosexuals as well, allowing the extension of the assessment of jealousy both for research purposes and for clinical assessment.

The results of the present work should also be considered in the light of a few limitations. Considering the large number of participants in the study, since they mostly resided in the regions of central Italy, it would be appropriate to extend the study to northern and southern regions in order to consider the influence of differences on the experience of jealousy due to socio-territorial specificity. In this regard future studies including quantitative and qualitative information are also needed to understand if the findings generalize to immigrant and ethnic groups which, due to cultural dissimilarities, could interpret the items in a different way (Alivernini et al., 2008, 2018, 2019; Milfont and Fischer, 2010). A further element to be considered is the mode of administration, which took place electronically and was entrusted to self-administration. In-person administration could have guaranteed greater attention and uniformity of the conditions of compilation. Considering the clear evidence obtained with the convergence analysis with measures of obsessive, paranoid, anxious and depressive jealousy, future studies could also include a clinical sample to assess the predictive ability of the instrument with respect to neurotic and psychotic delusional disorders, as described by the DSM-5 (American Psychiatric Association, 2013) and aggressive and dysfunctional behaviors within the love couple.

## Conclusion

The Italian validation study of the brief version of the multidimensional jealousy scale showed good results both for the CFA and for the measure of invariance across gender with an extended sample of participants. The three-factor model with five items each was confirmed to measure the cognitive, emotional and behavioral dimensions of jealousy. The values of internal consistency and convergent validity were good. This Italian version presents an adaptation in the formulation of the items that makes it possible to administer the scale also to non-heterosexual individuals. The scale is particularly suitable for quickly and efficiently collecting information on jealousy in a current relationship. The multidimensional and brief structure makes it particularly suitable for preliminary screening, couple therapy assessment and research purposes.

## Data availability statement

The raw data supporting the conclusions of this article will be made available by the authors, without undue reservation.

## Ethics statement

The studies involving human participants were reviewed and approved by the Institutional Review Board (IRB) of the University of Cassino and Southern Lazio. The participants provided their written informed consent to participate in this study.

## Author contributions

PD, GV, and SM designed the study, analyzed the data, and discussed the results. PD and LG drafted the manuscript. EC and AC revised the manuscript. All authors approved the final manuscript and agreed to be accountable for all aspects of the manuscript in ensuring that questions related to the accuracy or integrity of any part of it are appropriately investigated and resolved.
